# Electrical Potential and Cell Immobilisation Capacity of a Laser-Treated Titanium Alloy Surface

**DOI:** 10.3390/ma19061051

**Published:** 2026-03-10

**Authors:** Arturs Abolins, Alberta Aversa, Yuri Dekhtyar, Maris Dortins, Marks Gorohovs, Galina Khroustalyova, Lyubomir Lazov, Arturs Mamajevs, Mohammed Awad Hassan Olaish, Aleksander Rapoport, Elizabete Skrebele, Hermanis Sorokins, Edmunds Sprudzs

**Affiliations:** 1 Rezekne Academy of Technologies, Pils 23a, LV-4601 Rezekne, Latvia; arturs.abolins@rta.lv (A.A.); lyubomir.lazov@rtu.lv (L.L.); edmunds.sprudzs@rtu.lv (E.S.); 2 Department of Applied Science and Technology, Politecnico di Torino, Corso Duca degli Abruzzi, 24, 10129 Torino, Italy; alberta.aversa@polito.it; 3 Mechanical and Biomedical Engineering Institute, Riga Technical University, Kipsalas 6B, LV-1048 Riga, Latvia; jurijs.dehtjars@rtu.lv (Y.D.); marks.gorohovs@rtu.lv (M.G.); arturs.mamajevs@edu.rtu.lv (A.M.); m.mohammed-awad-hassan-olaish@edu.rtu.lv (M.A.H.O.); elizabete.skrebele@rtu.lv (E.S.); hermanis.sorokins@rtu.lv (H.S.); 4 Institute of Microbiology and Biotechnology, University of Latvia, Jelgavas Str. 1-537, LV-1004 Riga, Latvia; galinah@lanet.lv (G.K.); rapoport@mail.eunet.lv (A.R.)

**Keywords:** titanium alloy, colour, surface, electrical charge, yeast cell, immobilisation

## Abstract

Titanium and its alloys are widely used in endoprostheses. The naturally formed titanium dioxide film on titanium surfaces improves chemical stability and enhances implant biocompatibility. However, oxidised titanium surfaces may also promote bacterial adhesion and biofilm formation, contributing to implant-associated infections. Therefore, surface modification represents a key strategy for controlling microbial–implant interactions. This article focuses widely used titanium alloy Ti-6Al-4V treated with a laser beam, which induces surface colour changes as a result of oxide formation. Laser processing enables controlled formation of micro- and nanoscale features, structural reconstructions, and defects that may influence the surface electrical charge and, consequently, cell immobilisation. Thus, the surface colour, electrical potential, and cell immobilisation capacity are likely interrelated. From a manufacturing perspective, titanium oxide colouring facilitates quality control and process reproducibility, as surface colour provides a rapid, non-destructive visual indicator of oxide thickness and treatment consistency. This study aims to identify correlations among surface colour, electrical potential, and cell immobilisation capacity on laser-treated titanium alloys. A relationship between the optical properties, electronic structure, and biological response of laser-processed titanium oxide films is established. Specifically, the blue colour saturation of the oxide film is inversely correlated with the electron work function. A more saturated blue corresponds to a lower work function, indicating a higher positive surface charge density. This shift is attributed to changes in electron affinity, likely resulting from laser-induced structural reconstruction and defect formation within the oxide layer. The proposed changes in electronic structure are supported by modifications in the electronic density of states, analysed using near-threshold photoelectron spectroscopy. The biological response is directly linked to these physical changes: enhanced immobilisation of yeast (*Saccharomyces cerevisiae*) cells on the treated alloy surface correlates with the electron work function. These results may assist in the development of controlled titanium oxide surfaces with enhanced biocompatibility.

## 1. Introduction

Titanium alloys (TA) are widely used for endoprostheses because of their high strength-to-weight ratio, low density, and excellent corrosion resistance [1]. The naturally formed titanium dioxide (TiO_2_) film on surfaces enhances chemical stability and improves implant biocompatibility [2]. Furthermore, a surface of TA promotes favourable cell adhesion and osseointegration, which are critical for long-term implant performance [3].

However, TA may also facilitate bacterial adhesion and biofilm formation, contributing to peri-implant infections and implant failure [4]. Consequently, controlled surface modification is essential for regulating both cell–implant and bacteria–implant interactions.

Key surface characteristics influencing biological response include surface roughness, wettability, chemical composition, and electrical charge. These parameters strongly affect protein adsorption, cell adhesion, proliferation, and differentiation [5,6].

Among various surface modification techniques—such as plasma spraying, anodisation, chemical etching, and coating deposition—laser surface treatment has attracted particular attention. Laser processing enables precise, localised, and contamination-free modification of titanium surfaces, allowing controlled formation of micro- and nanoscale features without altering bulk material properties [7]. Such hierarchical structures may enhance, for instance, osteoblast attachment while simultaneously reducing bacterial colonisation, resulting in multifunctional surfaces desirable for implant applications [8].

A distinctive outcome of laser treatment is the controlled formation of coloured titanium oxide layers [9]. The colour mainly depends on oxide thickness due to optical interference effects [9,10,11,12]. Thinner oxide layers produce colours corresponding to shorter wavelengths [9]. Importantly, this colouration is not merely aesthetic but reflects changes in surface chemistry, morphology, and electronic structure. Variations in oxide thickness, crystallinity, and defect density influence surface morphology [13], the density of electrically active centres [14], and the oxide band gap [15], all of which govern surface electrical potential. Additionally, laser-induced modifications in surface roughness influence surface charge distribution [16].

Thus, surface colour, electrical potential, and biological response are intrinsically interconnected. Variations in oxide thickness and electronic structure influence surface electrical charge, which plays a central role in protein adsorption, cell adhesion, proliferation, and differentiation. Controlled titanium oxide colouring, therefore, enables tuning of implant surface properties to enhance osseointegration while potentially limiting bacterial adhesion and biofilm formation. Dense and uniform coloured oxide layers may also improve corrosion resistance and chemical stability, thereby extending implant longevity.

From a manufacturing perspective, titanium oxide colouring facilitates quality control and process reproducibility. Surface colour serves as a rapid, non-destructive visual indicator of oxide thickness and treatment consistency.

Despite its importance, the fundamental relationships between surface colour, electron work function (characterising the electrical charge density), and cell immobilisation remain insufficiently understood.

This study aims to elucidate these correlations for laser-treated Ti-6Al-4V surfaces.

## 2. Materials and Cells

Titanium alloy (Ti-6Al-4V) specimens measuring 10 × 10 mm were prepared by cutting stock material supplied by Taizhou Yutai Metal Materials Co. (Taizhou, China) using a Suntop ST-FC1390 fibre laser cutting machine (Suzhou Suntop Laser Technology Co., Ltd., Suzhou, China) under ambient atmospheric conditions. After cutting, the samples were rinsed with technical-grade ethanol, dried at room temperature, and subsequently subjected to laser surface treatment.

The experiments were conducted using the yeast *Saccharomyces cerevisiae* (baker’s yeast) as a model organism due to its high growth efficiency, cost-effectiveness, and ethical suitability for studying fundamental biological processes analogous to those occurring in human cells [17]. The immobilisation behaviour of *Saccharomyces cerevisiae* is strongly influenced by surface electrical charge density [18].

The strain *Saccharomyces cerevisiae 77* was obtained from the Microbial Strain Collection of Latvia. Yeast cultivation was conducted in 100 mL Erlenmeyer flasks containing 20 mL of growth medium. Two media were used: (i) YPD broth consisting of yeast extract (10), peptone (20), and glucose (20), and (ii) a salt-supplemented medium based on Reader’s formulation [19] containing (g L^−1^) MgSO_4_ (0.7), NaCl (0.5), (NH_4_)_2_SO_4_ (3.3), KH_2_PO_4_ (1.0), K_2_HPO_4_ (0.13), glucose (20), and yeast extract (3). The pH of both media was adjusted to 5.5 using KOH prior to autoclaving at 1 atm for 15 min.

Cultivation was performed at 30 °C for 24 h. The resulting biomass was harvested by centrifugation at 3000 rpm for 10 min using a benchtop centrifuge (Sigma 1-14, Sigma, Steinheim am Albuch, Germany). After removal of the supernatant, the pellet was resuspended in distilled water and adjusted to an optical density of OD_600_ = 0.5 using Helios Gamma UV–Vis spectrophotometer (Thermo Fisher Scientific, Waltham, MA, USA).

The prepared cell suspensions were dispensed onto laser-treated titanium alloy samples pre-cleaned with 96% ethanol and placed in sterile borosilicate Petri dishes (90 mm). Borosilicate glassware was used to minimise potential leaching of impurities or reactive ions that could affect pH or cell viability. To ensure uniform contact between yeast cells and the alloy surface, the Petri dishes were placed on an orbital shaker (ES-20/60 Orbital Shaker-Incubator, Biosan, Riga, Latvia) and agitated at 90 rpm for 60 min at 25 °C.

After agitation, the samples were removed using a vertical lifting motion to avoid tilting or contact with the dish walls, thereby preserving the natural distribution of cells and avoiding artefacts caused by fluid runoff. The specimens were then dried in a precision-controlled drying chamber (SNOL 58/350, AB “Umega Group”, Utena, Lithuania) at 30 °C for 48 h.

The prepared specimens were subsequently examined by optical microscopy to evaluate the extent of cell immobilisation on the titanium surfaces.

## 3. Methods

### 3.1. Laser Processing

Titanium dioxide formation was induced using a Rofin Power-Line F20 Varia fibre laser (Rofin-Sinar, Bergkirchen, Germany) operating at a wavelength of 1064 nm and a maximum output power of 20 W. The laser was operated at a pulse frequency of 700 kHz with a pulse duration of 4 ns.

The laser beam was scanned across the specimen surface at a speed selected to produce an effective treated line width of approximately 10 µm. A bidirectional scanning strategy was employed, alternating between left-to-right and right-to-left passes. Successive scan lines were oriented perpendicular (90°) to one another without overlap. Each processing area measured 10 × 10 mm and was treated using a single scan.

All treatments were performed under ambient atmospheric conditions. After processing, the samples were wrapped in aluminium foil and stored in sealed plastic bags at room temperature for three days prior to further characterisation.

By varying the laser processing parameters, distinct groups of specimens exhibiting different surface colours were obtained. All specimens within a given group were processed using identical parameters.

### 3.2. Surface Characterisation 

#### 3.2.1. Surface Colouration Analysis

Surface colour was characterised using the RGB (red–green–blue) colour model as a rapid method for indicating the colours [20] of thin metal oxide/metal systems [21]. This approach is well suited for titanium oxide [22] and, therefore, was used to assess the relationship between colour components for each specimen. Optical micrographs were acquired using a Carl Zeiss Jen NU 2 optical microscope (VEB Carl Zeiss Jena, Jena, Germany) at 50× magnification. Identical imaging conditions were applied to all samples.

To minimise the influence of local defects and imaging artefacts, images were post-processed using Adobe Photoshop (Version 26.10; Adobe Inc., San Jose, CA, USA). Gaussian smoothing was applied to suppress high-frequency components associated with scratches and micro-defects while preserving large-scale colour information.

A filter radius of 130 px was selected as the minimum value required to achieve adequate suppression of defect-related artefacts. Smaller radii were found to be insufficient.

RGB values were extracted from the central region of each scanned area using the software as above (Adobe Photoshop, Version 26.10; Adobe Inc., USA).

#### 3.2.2. Scanning Electron Microscopy and EDS 

Surface morphology and chemical composition were analysed using a TESCAN S9000 scanning electron microscope (TESCAN, Brno, Czech Republic), operated in secondary electron (SE) imaging mode and energy-dispersive X-ray spectroscopy (EDS) mode.

#### 3.2.3. Near-Threshold Photoelectron Spectroscopy

Near-threshold photoelectron spectroscopy (PES) was employed to investigate treatment-induced changes in surface electronic properties over millimetre-scale areas. The photoemission current (*I*) was measured in the vicinity of the emission threshold. To a first approximation, the magnitude of *I* is proportional to the electron work function (EWF), which represents the energy required for an electron to escape from the surface.

The EWF is defined as the energy difference between the vacuum level and the highest occupied electronic state. As it is known, the EWF is directly related to the surface electrical charge density: higher EWF values correspond to greater negative surface charge density.

Because the EWF of Ti and its oxide is around 4.3–5.8 eV, [19,23], the specimens were irradiated with photons of energy (*hν*) ranging from 4 to 6 eV. The EWF was determined by extrapolating *I*(*hν*) to zero photoemission current. Measurements were performed under high-vacuum conditions using a custom-built spectrometer [24]. The uncertainty in EWF determination was approximately 0.2 eV [25].

#### 3.2.4. Atomic Force and Kelvin Probe Force Microscopy

Surface roughness and local electrical potential were measured using a Solver Pro NT-MDT scanning probe microscope (NT-MDT Spectrum Instruments, Moscow, Russia).

HA_NC/W2C cantilevers (TipsNano, Tallinn, Estonia) were used for both AFM and KPFM measurements. In KPFM mode, the Kelvin potential was determined as the difference (Δ) between the electron work function of the specimen surface and that of the tungsten carbide-coated, electrically conductive cantilever [26]. The potential difference Δ is defined according to Equations (1)–(3):

(1) $$\Delta =\varphi_{s}-\varphi_{c}$$

where *φ_s_* and *φ_c_*—electron work functions of the specimen and cantilever, respectively [26],

(2) $$\varphi_{s}=\chi +F$$

where *χ*, *F*—electron affinity of the specimen and its Fermi energy,

(3) $$\varphi_{c}=\chi_{c}+F_{C}$$

where *χ_c_*, *F_c_*—electron affinity of the cantilever and its Fermi energy counted from the bottom of the conduction band energy. Both *χ_c_* and *F_c_* have constant values as the cantilever is not processed during the experiment.

Surface roughness and electric potential distributions were recorded using the Nova software package (version 1.1.0.1921; NT-MDT Spectrum Instruments, Russia). Each scan covered an area of 10 × 10 µm^2^ with a spatial resolution of 256 × 256 data points. For each specimen, five scans were acquired at randomly selected locations on the surface.

Subsequent image processing and filtering were performed using the Gwyddion software (version 2.68; Czech Metrology Institute, Brno, Czech Republic). This processing enabled the extraction of surface electric potential data and their export as text files for further analysis.

The surface roughness of the specimens, characterised by the arithmetic mean height parameter *S_a_*, was found to lie in the range of 20–60 nm.

#### 3.2.5. Film Thickness and Colour

The relationship between oxide film thickness and colour was attributed to optical interference effects [9]. Specimens were illuminated using a 60 W tungsten filament lamp positioned 50 cm perpendicular to the surface. According to the results reported in [27], the films exhibit purple, green, and red colours when their thicknesses are approximately 114, 146, and 179 nm, respectively. Consequently, thinner films shift the observed colour toward shorter wavelengths (blue region).

#### 3.2.6. Data Processing

Data was processed using Microsoft Excel 365 (Microsoft, Redmond, WA, USA) and OriginPro 2018 (OriginLab Corporation, Northampton, MA, USA). Outliers and linear correlations were identified using a 95% confidence level [18,28].

To detect outliers within treatment groups, R–G diagrams were constructed. Data points deviating significantly from the group mean at a confidence level of 95% were excluded from further analysis.

### 3.3. Analysis of Immobilised Cells

After cell deposition, optical microscopy (NU-2, Carl Zeiss Jena, Jena, Germany) was performed at 200× magnification to quantify surface coverage. A grid-based imaging strategy was used: 25 micrographs per sample were acquired using a 5 × 5 square grid with approximately 1.5 mm spacing between nodes.

Image analysis was conducted using a combination of MicroSAM (version 1.2), and ImageJ (version 1.54r) software. MicroSAM a microscopy-adapted extension of Meta AI’s Segment Anything Model, is a deep-learning-based segmentation tool trained on a large dataset of microscopy images to identify and distinguish cell-like structures from the background. Unlike conventional thresholding or edge-detection methods, MicroSAM employs a prompt-based artificial intelligence approach that demonstrates robust performance across varying image types, illumination conditions, and surface textures [29].

Following segmentation, the resulting binary masks were exported to ImageJ, an open-source image analysis platform [30], enabling high-throughput batch processing. The “Threshold” and “Analyze Particles” functions were used to calculate the fraction of the surface area covered by cells, expressed as a percentage of the total imaged area.

Several validation steps were incorporated into the image-processing workflow. First, images exhibiting visual artefacts (e.g., under- or over-focus, motion blur) or contamination by non-cellular particulate matter were excluded from analysis. Images affected by imaging artefacts were reacquired, whereas contaminated images were discarded. Second, the accuracy of automated segmentation was verified by comparing the results with manually hand-traced cell areas. Thresholding parameters were subsequently refined to achieve a marking accuracy exceeding 95%.

## 4. Results and Discussion

### 4.1. Results

**Figure 1:**
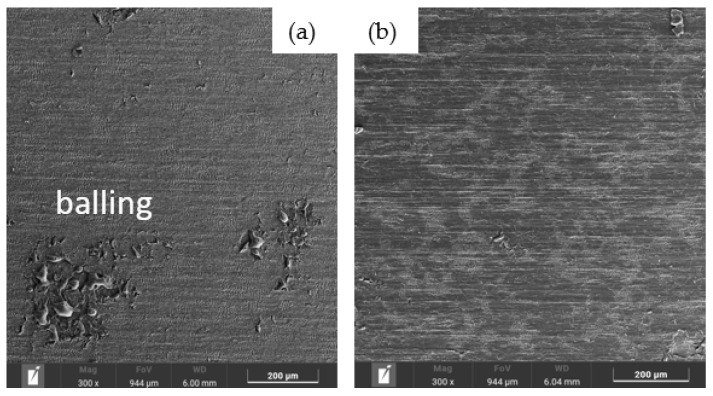
Influence of the laser beam scanning velocity on balling formation: (**a**) 80 mm/s; (**b**) 160 mm/s.

Figure 1 presents an example of the SEM image.

Observations of obtained images revealed that decreasing the laser beam scanning velocity led to the formation of small spherical features (balling) on the specimen surface. Figure 1a shows the presence of balling at a scanning velocity of 80 mm/s, whereas Figure 1b demonstrates that balling was not observed at the higher velocity of 160 mm/s.

**Figure 2:**
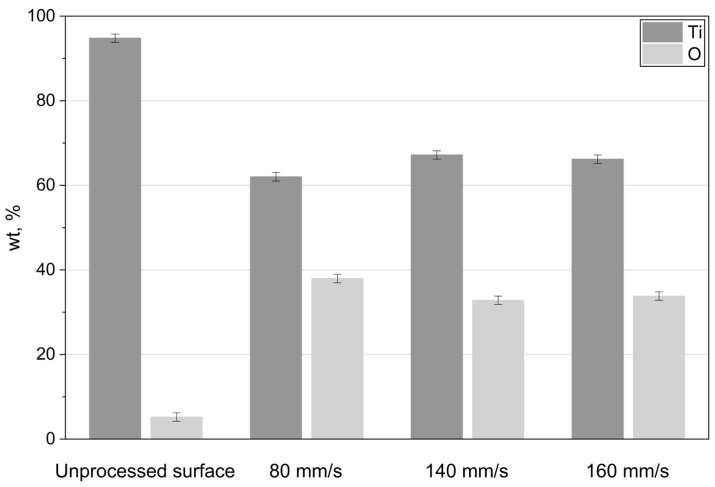
Titanium and oxygen weight percentages as a function of the laser beam scanning velocity.

Figure 2 shows that the chemical composition of the fabricated titanium oxide films does not significantly depend on the laser beam scanning speed.

A slight increase in oxygen concentration was observed at the lowest scanning velocity of 80 mm/s.

EDS analysis indicated that the oxide layer was close to the stoichiometric composition of TiO_2_. The measured Ti:O ratio was approximately 60/(35–40) wt%, which is consistent with the theoretical stoichiometric ratio of 60/40 wt%.

Based on these results, subsequent specimen processing was performed at the higher scanning velocity.

**Figure 3:**
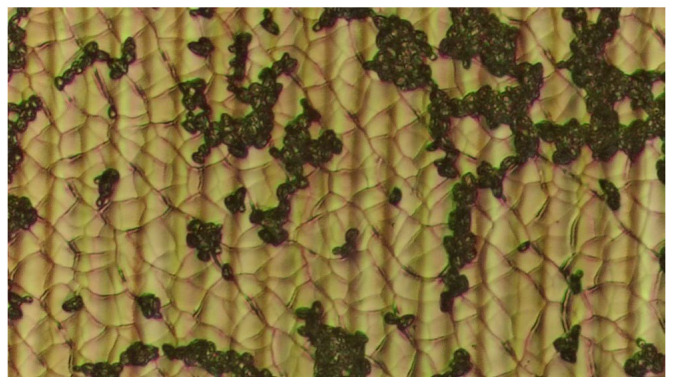
Optical micrograph of the yellow-coloured laser-treated Ti-6Al-4V surface after yeast cell immobilisation (magnification 200×).

**Figure 4:**
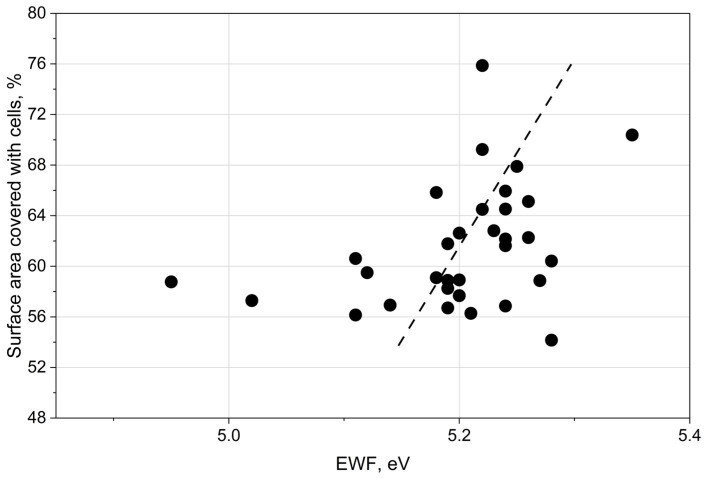
Fraction of the laser-processed surface area covered by cells as a function of the electron work function (EWF).

The fraction of the laser-processed surface covered by cells (an example is shown in Figure 3) exhibited a positive correlation with the electron work function (EWF), as presented in Figure 4.

The observed result is consistent with previous findings [29], which demonstrated that yeast cells exhibit enhanced immobilisation on negatively charged surfaces.

**Figure 5:**
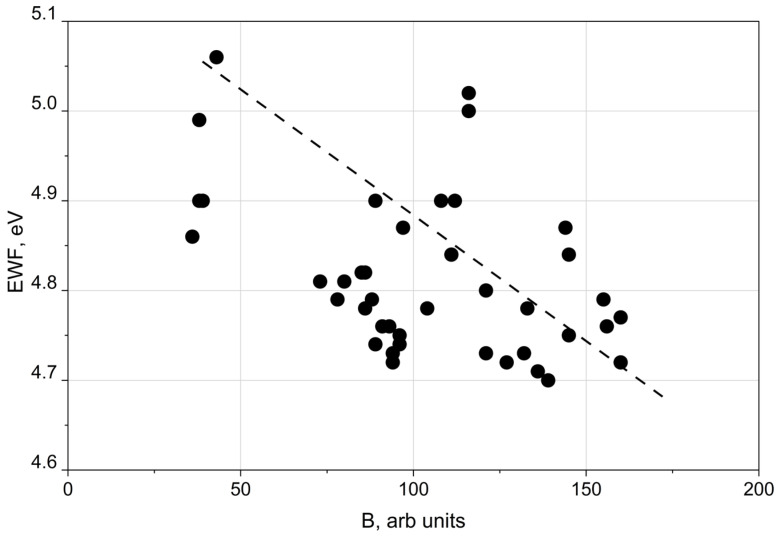
Relationship between the electron work function (EWF) and the blue (B) colour component.

The influence of the EWF on cell immobilisation also correlated with the colour of the processed surface. Figure 5 presents that the strongest correlation was observed for the blue (B) colour component.

**Figure 6:**
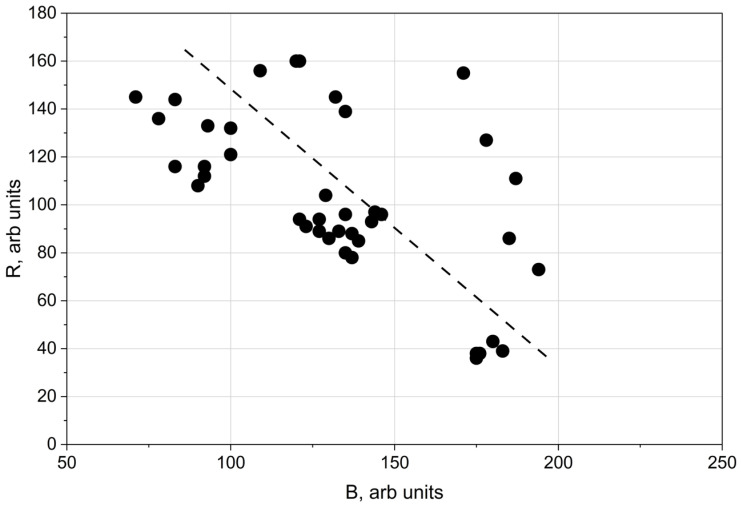
Relationship between red (R) and blue (B) colour components of the laser-treated surfaces.

Figure 6 further demonstrates that an increase in the blue component was accompanied by a corresponding decrease in the red (R) component.

**Figure 7:**
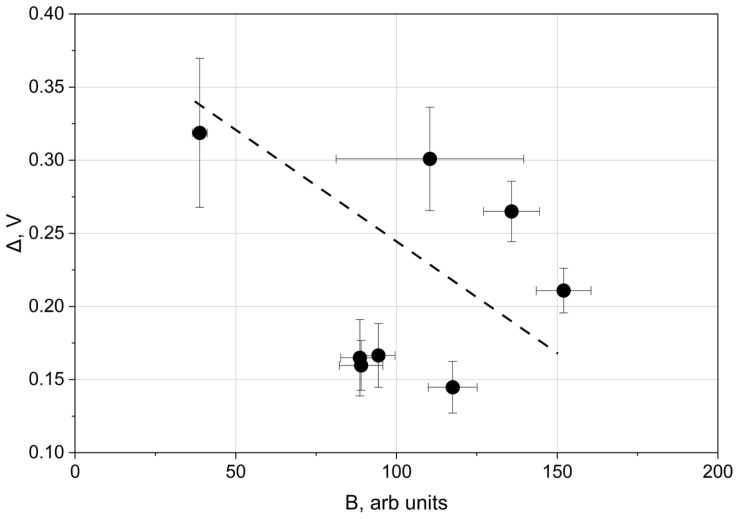
Kelvin potential (KP) as a function of the blue (B) colour component for different laser-processed specimen groups.

These observations motivated direct measurements of the surface electrical potential as a function of the blue colour value (B) to verify whether surface colour reflects electrical properties. As shown in Figure 7, the Kelvin potential decreased with increasing B.

Therefore, the blue colour component may serve as an indicator of the electron work function. The results presented in Figure 4 and Figure 5 support the conclusion that oxide film thickness influences the electron work function. Figure 7 demonstrates that the Kelvin potential exhibited a similar correlation with the blue colour component as observed for the EWF.

### 4.2. Discussion

The correlation shown in Figure 4 is consistent with the findings reported in [15], which demonstrated that yeast cells exhibit enhanced immobilisation on negatively charged surfaces characterised by higher EWF values.

The relationship between the electron work function (EWF) and the blue (B) colour component can be explained by the dependence of surface colour on oxide film thickness [12]. Thinner oxide films preferentially satisfy interference conditions for shorter wavelengths [12], resulting in increased blue saturation. Conversely, an increase in the blue component is accompanied by a reduction in the red component, as confirmed by the data in Figure 6. The results presented in Figure 5 indicate that decreasing oxide thickness—reflected by increased blue colour intensity—is associated with a decrease in the electron work function. The Kelvin potential exhibits a comparable correlation with the blue colour component, as shown in Figure 7.

According to Equations (1)–(3), and considering that the cantilever parameters remain constant, a reduction in the Kelvin potential difference (Δ) may result from either a decrease in electron affinity (χ) or a shift in the Fermi-level position (F). A reduction in oxide thickness is known to increase the band gap (Eg) [15]. Since the fabricated oxide films are non-crystalline and contain a high density of structural defects [31], which act as charge traps [31,32], the Fermi level is expected to be pinned near the mid-gap region. Therefore, F ≈ Eg/2, and bandgap widening with decreasing oxide thickness is unlikely to significantly reduce Δ through Fermi-level shifts alone.

Consequently, the observed reduction in Δ is most plausibly associated with a decrease in the electron affinity *χ.* Such changes in *χ* may arise from laser-induced structural reconstruction and defect formation within the oxide layer. If laser processing modifies the electron affinity through structural changes, corresponding alterations in the electronic density of states should be observable in the near-threshold photoemission spectra.

**Figure 8:**
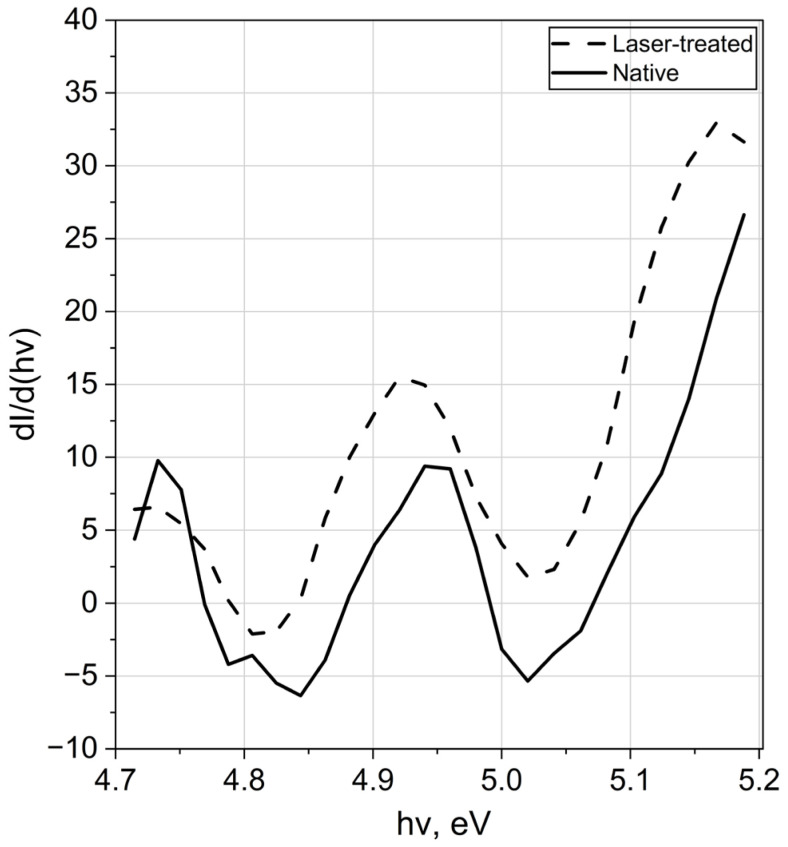
Near-threshold photoemission spectra of native and laser-treated Ti-6Al-4V specimens.

Indeed, laser-induced modifications of dI/d(hν) detected in the photon energy range of 4.7–5.2 eV are presented in Figure 8.

Laser treatment resulted in an increment (Δ*N*) in the integrated number of electronic states (*N*), defined as:

(4) $$∆N\sim \int_{4.7}^{5.2}\frac{dI}{d\left(hv\right)}d(hv)$$

**Figure 9:**
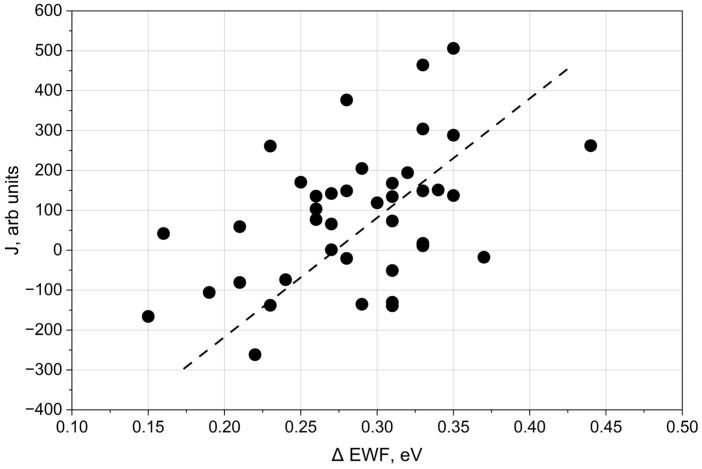
Dependence of the integral J on the change in electron work function (ΔEWF).

This increment is expected to correlate with the corresponding change in the electron work function. To verify this relationship, the correlation between the integral (*J*) (defined in Equation (4)) and ΔEWF was analysed in Figure 9.

The results in Figure 9 indicate that the number of electron emission centres generated by laser treatment increases with increasing EWF, which corresponds to reduced blue colouration of the processed surface. Accordingly, the observed decrease in Δ with increasing blue colour intensity (B), associated with decreasing oxide thickness, is attributed primarily to changes in electron affinity rather than Fermi-level shifts.

## 5. Conclusions

1. This study establishes a relationship between the colour, electronic structure, and biological response of the laser-processed titanium oxide films formed on Ti-6Al-4V alloy. The blue colour saturation of the oxide film is inversely correlated with its electron work function. This energetic shift is attributed to laser-induced structural reconstruction and defect formation within the oxide layer, which modifies the electronic structure. These changes are supported by alterations observed in the electronic density of states in the 4.7 and 5.2 eV range, as revealed by near-threshold photoelectron spectroscopy.
2. The biological response of the surface is directly linked to these electronic modifications. The immobilisation efficiency of *Saccharomyces cerevisiae* cells on the treated alloy surface increases with the growth of the electron work function, demonstrating that laser-induced electronic structure changes influence cell–surface interactions.
3. The demonstrated correlation between surface colour, electron work function, and yeast immobilisation efficiency enables rapid optical assessment and predictable tuning of titanium oxide surface properties. This approach represents a controllable and chemical-free strategy for functional surface engineering with potential applications in biomedical and biotechnological materials design.

## Data Availability

The original contributions presented in this study are included in the article. Further inquiries can be directed to the corresponding author.
